# Correction to PV1 downregulation via shRNA inhibits the growth of pancreatic adenocarcinoma xenografts

**DOI:** 10.1111/jcmm.17980

**Published:** 2023-11-02

In Deharvengt, et al,^1^ a technical error has occurred during the assembly of panel 4A in Figure 4. The correct Figure 4A is shown below in the corrected Figure 4. All results and conclusions of this article remain unchanged.

Additionally, we have become aware that the control tumour growth data for the BxPC‐3 line in Figure 2B may have been published in another paper by the collaborating lab where these data were obtained. While we were not able to verify this conclusively, out of abundance of caution, we have decided to remove the data obtained with the BxPC‐3 cell line from this article. Figures 2 and 4 and their legends were modified to remove the data obtained with the BxPC‐3 line. The corrected Figures 2 and 4 and their respective Figure Legends are shown below. The article text has been modified to remove references to the BxPC‐3 cell line. As the results obtained with BxPC‐3 cell line and AsPC‐1 cell line are similar, this action does not change the conclusions of this paper.

**FIGURE 2:**
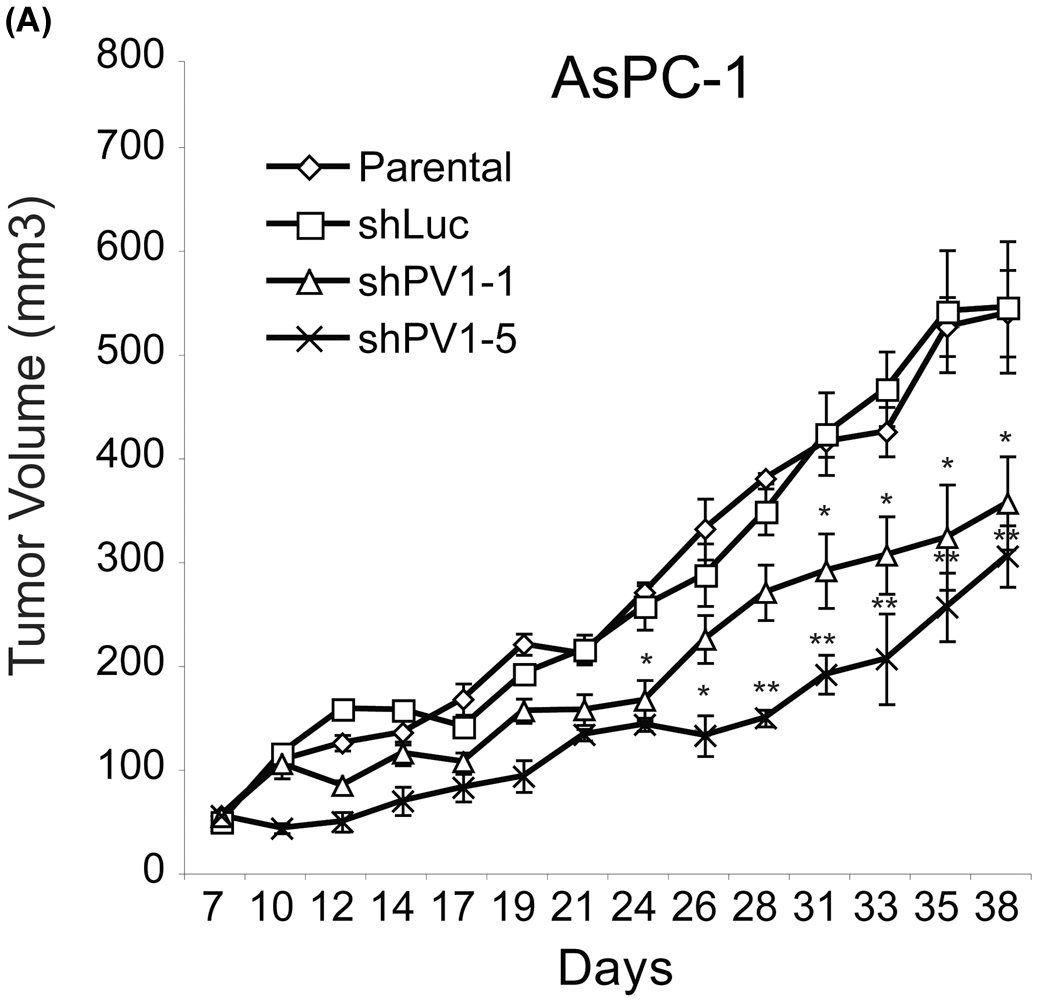
Intratumoral delivery of PV1 shRNA inhibits pancreatic tumour growth. Growth curves of AsPc‐1 (A) tumours either non‐treated (open diamonds) or injected with shLuc‐LV (open squares), shPV1‐1‐LV (open triangles) and shPV1‐5‐LV (x). (*n* = 8 per group, *p* < 0.01, error bars – SEM).

**FIGURE 4:**
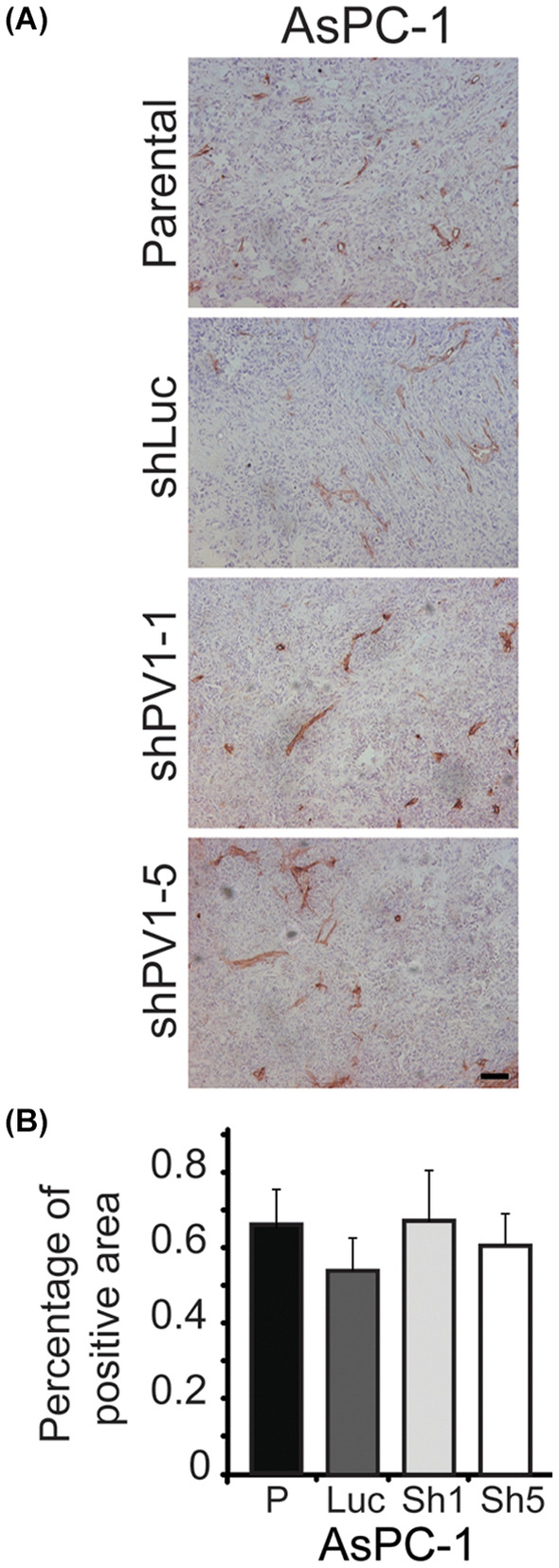
Downregulation of PV1 does not decrease tumour vascular density **(**A) Immunohistochemistry with anti‐CD31 antibodies of AsPC‐1 tumours untreated (parental) or treated with shLuc‐LVs (shLuc), shPV1‐1‐LV (shPV1‐1) and shPV1‐5‐LV (shPV1‐5). (B) Morphometric analysis of vascular density in AsPC‐1 tumours (shLuc‐LVs (Luc), shPV1‐1‐LV (sh1) and shPV1‐5‐LV (sh5)). Error bars – SEM.

The corrections to the article text are as follows:

In the Abstract, the fourth sentence should read: ‘We find that PV1 downregulation by shRNAs inhibits the growth of established tumours derived human pancreatic adenocarcinoma AsPC‐1 cell line’.

In the Methods section, under subheading ‘Pancreatic tumour xenograft model’ the first sentence should read: ‘Female athymic mice (Nu/Nu, Charles River) were injected subcutaneously into the dorsal flank area with 1 × 10^6^ of ASPC‐1 cells’. Under subheading ‘Colocalization of PV1 and CD31 in tumour samples by confocal microscopy’, the first sentence should read: ‘AsPC‐1 tumours were induced as described and allowed to grow for 21 days’.

In the Results Section, under subheading2 Intratumoral delivery of PV1 shRNA inhibits the growth of established pancreatic tumours’, the first sentence should read: ‘To determine whether the lentivirus mediated PV1 downregulation is able to inhibit PDAC growth we employed a model system consisting of heterotopic tumours derived from human PDAC cell line AsPC‐1 grown subcutaneously in nude mice’. Second sentence should read: ‘Following subcutaneous injections of AsPC‐1 cells, tumour growth was monitored and mice with established tumours (volume of 50 mm^3^) were enrolled in four different groups, one to be left untreated and three treated with one injection either of shPV1‐1‐LV, of shPV1‐5‐LV or shLuc‐LV’. The fourth sentence should read: ‘A statistically significant difference in tumour volumes between shLuc‐LV‐injected tumours and shPV1‐LV‐injected tumours was seen as early as 24 and 33 days following viral injection of AsPC‐1‐derived tumours’. Seventh sentence is deleted. Last sentence should read: ‘Taken together, these data demonstrate that PV1 downregulation by two different shRNAs are able to inhibit pancreatic tumour growth in human PDAC xenograft models’.

Under subheading3 PV1 is expressed specifically in the endothelial cells of tumour xenografts and is not expressed in tumour cells’, the second and third sentences should read: ‘To strengthen this observation, we defined the precise cellular type(s) expressing PV1 in the AsPC‐1 derived tumours. Although PV1 is expressed in tumour ECs in many solid tumours including pancreatic cancer, there is no previous information regarding PV1 expression in AsPC‐1 derived tumours’. The last two sentences should read: No expression of human PV1 was detected in the AsPC‐1 or BxPC‐3 tumour sections. Thus, in AsPC‐1 tumours, PV1 is specifically expressed in the tumour ECs and not in the tumour cells per se, in good agreement with data in the literature obtained in other tumours.

Under subheading4 PV1 downregulation does not affect vascular density of tumours’ the fourth sentence should read: ‘Quantitation data show that there are no statistically significant differences in vascular area density between the control and PV1 shRNAs treated AsPC‐1 derived tumours (Figure 4B)’.

In the Discussion Section, under paragraph three, the second sentence should read: ‘We used tumours derived from AsPC‐1 cells, an established human pancreatic ductal adenocarcinoma cell line, isolated from metastatic PDAC tumours’.
